# Physical Characterization of Multiwire Polystyrene Produced by Electrospinning Technique

**DOI:** 10.3390/polym17192587

**Published:** 2025-09-24

**Authors:** Lorenzo Torrisi, Letteria Silipigni, Alfio Torrisi, Mariapompea Cutroneo, Angela Malara, Antonio Fotia, Chiara Nunnari, Patrizia Frontera

**Affiliations:** 1 Dipartimento MIFT, Università di Messina, Viale F.S. d’Alcontres 31, 98166 Messina, Italy; ltorrisi@unime.it (L.T.);; 2 Dipartimento di Medicina e Chirurgia, Università di Enna “Kore”, 94100 Enna, Italy; alfio.torrisi@unikore.it; 3 Department of Civil, Energetic, Environment and Material Engineering, Mediterranean University of Reggio Calabria, 89124 Reggio Calabria, Italy; 4 CNR-ITAE, Istituto di Tecnologie Avanzate per l’Energia, 98126 Messina, Italy

**Keywords:** polystyrene, multiwire polystyrene, optical spectroscopy, SEM morphology, wetting measurements, permittivity measurements

## Abstract

Multiwire polystyrene (PS) produced by the electrospinning technique was physically characterized in terms of morphology and by optical properties of transmittance and absorbance in the IR and UV-Visible regions. A comparison was presented with the properties of bulk PS and multiwire PS containing graphene oxide (GO) nanoparticles (NPs). The polymer is hydrophobic, and this surface property is enhanced when it has a multiwire morphology, and even more when GO NPs are embedded in it. The wetting angles reach up to about 144°. PS is an optimum dielectric polymer, and its permittivity was measured as a function of frequency. Some possible applications of the produced multiwire PS are presented and discussed.

## 1. Introduction

Biocompatible polymers have been increasingly requested to create devices that can be used in various scientific fields, such as biology, medicine, engineering, food processing, filtering of liquids, gases, and solids, sensors, microelectronics, preparation of prostheses, and more [1]. There is an increasing demand for innovative and intelligent polymeric and composite materials, such as micro- and nanomembranes for special uses as sensors for various parameters, such as specific gases, temperature, humidity, pressure, and electrical quantities, with selective optical, mechanical, electrical, and thermal properties, as well as special chemical behaviors [2].

In particular, polymers with high water, biological liquid, hydrogen, oxygen, and other gas absorbance, as well as microparticles and nanoparticles of various types, are in high demand for diverse applications. Moreover, membranes with selectable filtering of water, gases, ions, microparticles, and controllable diffusivity of toxic and non-toxic gases are requested [3]. Furthermore, thin polymeric films are used to realize resistant barriers [4] for optical filters, for biological applications, and for medical applications, such as substitutes for human skin, smart membranes that release drugs only when needed, accumulators of oxygen molecules, and more [5].

A separate discussion must be made for polymers designed to be highly absorbent to liquids, gases, and micro and nanoparticles, which can be used for water desalination, as absorbers of atmospheric particulate matter, as absorbers of toxic gases and pollution, as absorbers of liquids inside a human organ, or as sensors that can be controlled electronically, thermally, by solution pH, by pressure, or by other parameters [6].

In this scenario, nanofibers have gained the attention of the scientific community due to their versatility across tissue engineering [7], drug delivery [8], and as absorbers [9]. Nanofibers date back to 1747, even though the patent was documented in the 1900s [10].

Depending on the desired morphologies and features required for the application of interest, different techniques have been employed to synthesize nanofibers, from template synthesis to electrospinning. The latter [11] emerged as the most practical technique for the production of nanofibers. Despite the simplicity of the processing, various factors, including molecular weight, viscosity, polymer concentration, used solvent, applied voltage, and flow rate, affect the size, morphology, and crystalline/amorphous nature of the final product.

Electrospinning allows for the realization of hybrid multiwire polymeric microfibers with high absorbent properties. This method is based on the use of electrical forces to draw charged threads of polymer solutions, producing nanofibers with wire diameters ranging from nanometers to micrometers [12,13,14].

Wannatong et al. [15] reported on the effectiveness of solvents such as DMF (dimethylformamide), having a higher density and boiling point, in the synthesis of fibers with a small diameter of about 430 nm, as well as the formation of a high number of beads when polymer and solvent have a high solubility gradient. Jarusuwannapoom et al. [16] established that only a couple of solvents have dipole properties and conductivity properties suitable for the electrospinning of polystyrene. Despite plenty of studies, the effective control and assessment of the correlation between increased voltage and decreased fiber diameter, as well as the jet instabilities occurring below or above the critical voltage, are complex and not fully accomplished [17].

Although PS-GO composites have been previously explored, there are relatively few studies that provide a comprehensive characterization of the resulting electrospun material. In this paper, our attention is devoted to multiwire polystyrene (PS) prepared by electrospinning with and without adding graphene oxide (GO) nanoparticles (NPs) [18,19]. Their chemical formulation is (C_8_H_8_)_n_, where n is the number of monomers. We have focused on offering detailed morphological, structural, and electrical analyses of the composite fibers without any functionalization or blending agents (like APTES or polymeric additives) [20], which we believe adds valuable insight to the existing literature. The C/H atomic ratio assumes a value of 1 in the pure polymer, but it increases when embedding GO NPs into the polymeric structure. GO has recently proved its versatility under laser and ion irradiation [21]. Radiation ionization, for example, may change the properties of the multiwire PS-GO composite, modifying its properties and making it usable for trace detectors, ionizing radiation detectors, or even as a small-sized dosimeters, in accordance with our collateral research works reported in the literature [22,23,24]. Now, it has been selected as a filler in PS, being a good candidate for the enhancement of the load transfer between matrix and filler to improve the native properties of PS. Moreover, the presence of oxygen functional groups improves both the GO dispersion in solutions, assisting the preparation of polymeric microfibers [25], and the interfacial bonding with polystyrene chains [26].

Methods for incorporating nanoparticles into the polymer matrix [27] could enhance the characteristics of both the host matrix and the nanoparticles embedded within it. Compared to bulk materials, nanoscale materials could exhibit remarkable physical and chemical capabilities due to their high surface-to-volume ratio, which allows for precise tuning. Among several polymers, polyvinyl alcohol (PVA), poly(methyl methacrylate) (PMMA), and polyvinyl chloride (PVC) have been widely investigated [28]. Nevertheless, the discourse surrounding polystyrene nanocomposites remains limited, above all regarding some of the physical properties, such as dielectric properties or the absorption of liquids.

## 2. Materials and Methods

Polystyrene (PS) (average Mw = 192,000), dimethylformamide (DMF), and graphene oxide powder (average number of layers, 15–20) were used for the production of fibers. All reagents were purchased from Merck Life Science S.r.l. (Milano, Italy).

### 2.1. Sample Preparation

The production of electrospun microfibers starts with the preparation of a polymeric solution, according to the following procedure: for polystyrene (PS) microfibers, the pellets were dissolved in DMF and stirred at 600 RPM for four hours at room temperature. To obtain the polystyrene–graphene oxide microfibers (PS-GO), the GO powder was added to DMF and put in an ultrasonic bath for five minutes, and then PS pellets were added to the solution and stirred under the same conditions used for PS microfibers. Polymeric solutions were first introduced into a 10 mL glass syringe equipped with a 0.7 mm metal needle for the electrospinning process. A pump was utilized to regulate the flow rate. The electrospinning procedure was conducted at 25 °C with a relative humidity of 40%. Table 1 summarizes the electrospinning parameters used to produce each sample.

**Figure 1:**
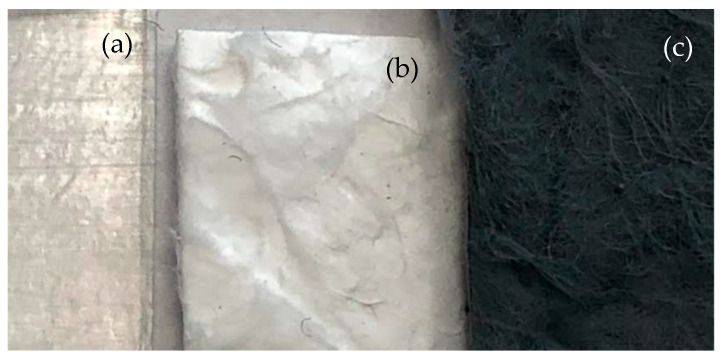
Optical images of PS bulk (**a**), PS filiform (**b**), and PS filiform with GO NPs (**c**).

The optical images of the investigated samples are shown in Figure 1.

### 2.2. Analysis Techniques

The prepared polymers have been investigated using Attenuated Total Reflectance (ATR) coupled to Fourier Transform Infrared (FTIR) spectroscopy. The infrared light travels through a diamond ATR crystal, is internally reflected, and such reflection travels to the FTIR detector. During the internal reflection, a part of the IR light travels into the sample, where it can be partially absorbed (evanescent wave). The absorption depends on the molecular composition of the sample and, as output, provides a characteristic IR spectrum. The penetration depth of IR into the sample depends on the refractive index difference between the sample and the ATR crystal, giving rise to transmittance or absorbance spectra. In our instrument (Jasco AT/IR 4600, Easton, MD, USA), the spectra can be computer-acquired in the 400–4500 cm^−1^ wavenumber range with a resolution of 4 cm^−1^.

UV-Visible optical spectroscopy was performed in the 250–850 nm wavelength region by using the Jasco V-750 (Easton, MD, USA) double-beam spectrophotometer. A high-sensitivity photomultiplier detector provides accurate and reproducible measurements for low- to high-concentration samples. For high-resolution measurements, the spectral bandwidth can be set as narrow as 0.1 nm.

Optical microscopy was used for preliminary observation of the prepared polymers and measurement of water wetting. Based on the sessile drop technique, the wetting angle of distilled water was measured at room temperature, 1 atm pressure, and 50% humidity, using drops of 2 μL volume deposited using a microsyringe on the polymer surface and controlling online the contact angle formed between the baseline of the droplet and the tangent at the droplet–air interface. The optical images with 8× magnification have been recorded.

The morphological characterization of electrospun microfibers was performed using scanning electron microscopy (SEM) (Phenom ProX, Thermo Fisher Scientific, Waltham, MA, USA). SEM permitted the use of energy-dispersive X-ray spectroscopy (EDS) to analyze the C and O image distributions in the investigated polymers, detecting their X-ray emission at 282 eV and 523 eV, respectively.

The electrical characterization of PS samples was performed by analyzing the frequency dependence of the real part ε_1_ of permittivity, the imaginary part ε_2_ of permittivity, and the loss tangent tan δ in the (10^3^ ÷ 10^6^) Hz range at room temperature using an Agilent 4284A (Hewlett-Packard (HP), Palo Alto, CA, USA) precision LCR meter [29]. The PS samples were sandwiched between two symmetric stainless-steel electrodes of the Keysight 16451B (Santa Rosa, CA, USA) dielectric material test fixture, and a 2 V peak-to-peak voltage was applied. The guarded electrode had an area of about 19.64 mm^2^.

## 3. Results and Discussion

Our first investigation concerned the polymer morphology without and with the embedded GO NPs. SEM investigations have shown a high degree of micrometric diameter wire density distributed in random directions. Their length is high, of the order of 0.1–1 mm.

**Figure 2:**
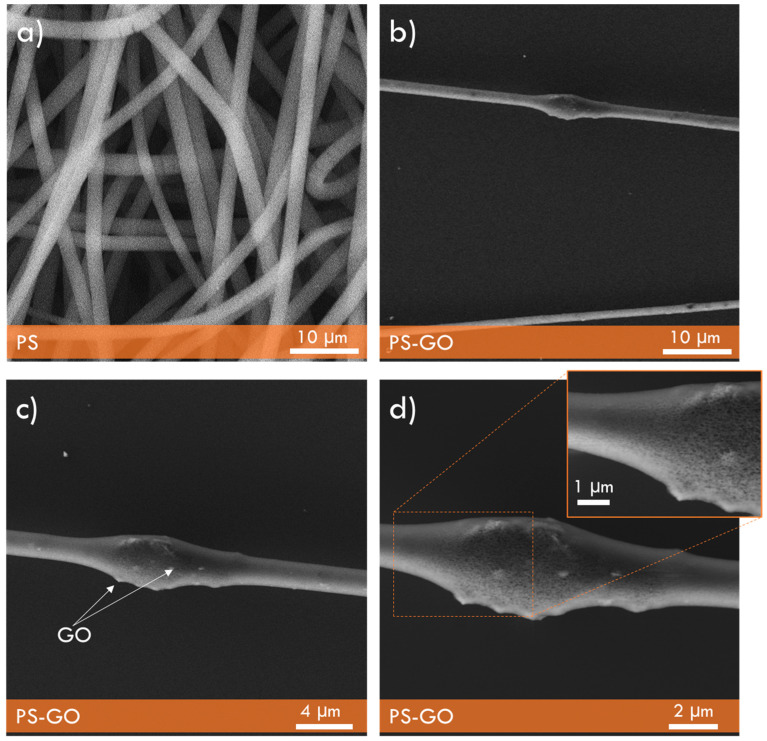
SEM image of pristine pure PS fibers (**a**) and of PS fibers with 10 wt% of GO NPs embedded into the polymer at different magnifications (**b**–**d**).

Figure 2a reports an SEM photo of the pristine pure PS fibers morphology at 5000× magnification, while Figure 2b focuses on two wires when GO NPs have been embedded at 10% by weight. Some beads are present along some wires. In this case, it is possible to observe the chemical attachment of the NPs, which are evident in the zoomed-in photos of Figure 2c,d. The images highlight an average wire diameter of about 1 μm and a doubling in diameter over a length of about 5 microns at the points where multiple GO NPs have nucleated on the PS. Spots are also randomly distributed along the wire surface, indicating the presence of single GO nanoparticles embedded in the polymer. The insert of Figure 2d shows a zoom of 20 k× magnification of the GO NPs in the PS wire.

**Figure 3:**
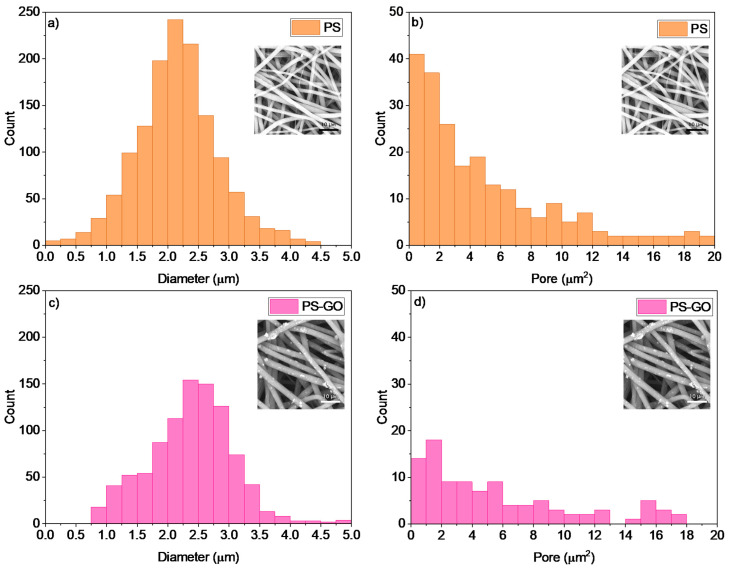
Wire diameter distributions in PS (**a**) and PS-GO (**c**), and pore size distribution in PS (**b**) and PS-GO (**d**).

SEM images have allowed the measurement of the distributions of wire diameters in electrospun PS without and with GO embedding at 10 wt%, as reported in the plots of Figure 3a and Figure 3c, respectively. The average diameter is 2.0 μm in PS and 2.5 μm in PS + GO. Moreover, they have given the pore size distributions of both PS and PS + GO multiwire polymers, as reported in Figure 3b and Figure 3d, respectively. The average pore size is 1.0 μm^2^ in PS and 2.0 μm^2^ in PS + GO, although the total pore size is reduced by about 50% in PS + GO concerning the PS.

**Figure 4:**
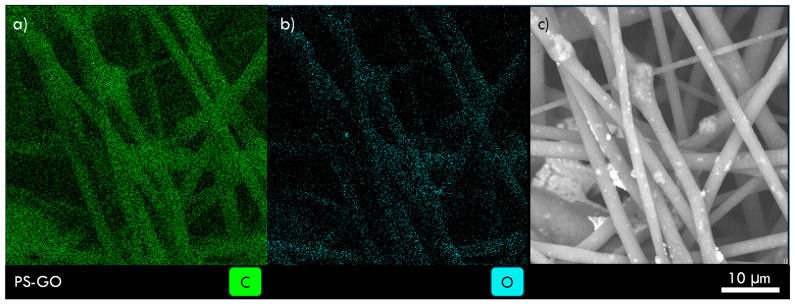
X-ray image of carbon distribution (**a**), oxygen distribution (**b**), and comparison with SEM image (**c**) of a PS-GO multiwire polymer.

EDS has permitted us to evince the carbon and oxygen distributions by monitoring the X-ray fluorescence emission induced by a 15 keV electron beam, as reported in the images of Figure 4. The carbon (a) and oxygen (b) maps are given in comparison with the SEM image (c) in a PS-GO multiwire polymer, indicating a high yield of carbon and a minor yield of oxygen, this last as functional groups of oxygen present in the embedded GO with a uniform distribution, as expected.

**Figure 5:**
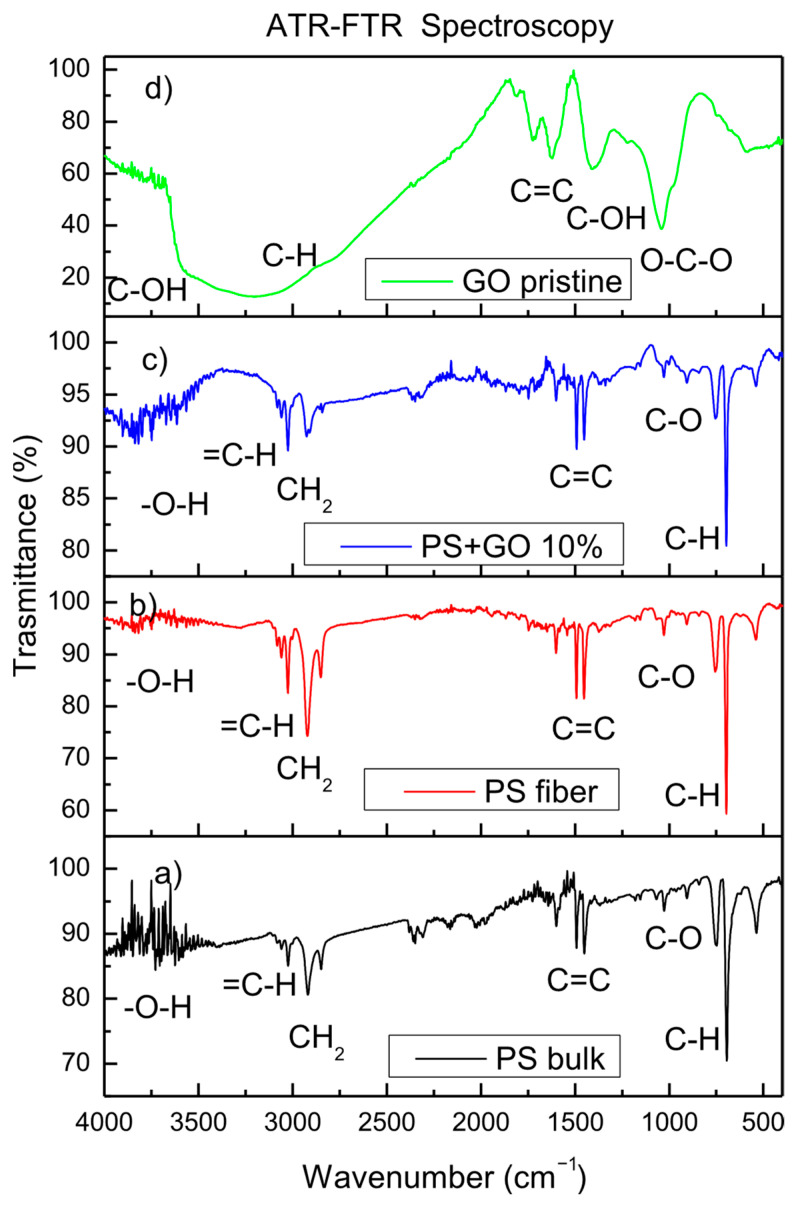
ATR-FTIR spectra comparison between PS bulk (**a**), PS filiform (**b**), PS filiform with GO NPs (**c**), and GO pristine (**d**).

A further analysis of the characterization of the synthesized PS multiwire polymer has been performed using ATR-FTIR spectroscopy. Figure 5 reports the FTIR spectra comparison between the PS bulk, the filiform electrospun PS, and the PS filiform with GO embedded as NPs at a 10 wt% concentration.

The pure and dense bulk PS FTIR transmittance spectrum shows characteristic bands at 3100–3000 cm^−1^, corresponding to the =C-H stretching due to the aromatic ring at 2924 and 2846 cm^−1^, due to the symmetrical and asymmetrical stretching vibration of CH_2_ at 1602, 1490, and 1447 cm^−1^, attributable to the stretching vibration of the C=C bond on the benzene ring. Bands at 1023 cm^−1^ correspond to C-O bonds, and bands at 902 cm^−1^ can be assigned to the C-H out-of-plane bending vibration of the benzene ring [30,31,32]. In the region of 3500–3900 cm^−1^, there are bands due to the hydroxyl groups -O-H. The bands of O-H stretching between 3700 and 3500 cm^−1^, and of H-O-H water bending deformation between 1650 and 1600 cm^−1^, attributed to atmospheric water vapor during measurements, were subtracted from the original spectra. The FTIR bands observed in the pure and dense bulk PS also characterize the filiform PS foil. In the multiwire fibers, the C-H and CH_2_ bands are more intense than in PS bulk, but the total transmittance is higher than in the bulk, due to its lower density, non-homogeneity and compactness, and higher porosity.

The presence of GO in filiform PS is low, evident from its low concentration of 10 wt%, but the presence of the different functional groups of oxygen (hydroxyl, carbonyl, carboxyl, epoxy, and water) is evident and has been researched in detail. Figure 5d reports a typical FTIR transmittance spectrum due to only GO. The most prominent features of the GO spectrum are the adsorption peaks at 3432 cm^−1^ and 1678 cm^−1^, which correspond to the hydroxyl groups (-OH) and carboxyl groups (-C(=O)OH), respectively. Furthermore, the peaks at 1770 cm^−1^ and 1071 cm^−1^ correspond to the stretching vibration of carbonyl (C=O) and the antisymmetric stretching vibration of the epoxy bond (-C-O-C), respectively [33].

The C-O stretching vibration peak in PS + GO is due to the oxygen functional groups present in GO nanoparticles; in multiwire PS, it is due to the gases absorbed between the wires, while its weak presence in PS could be due to surface contamination of the polymer.

**Figure 6:**
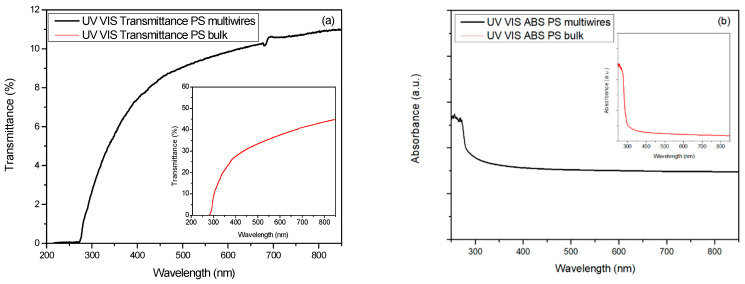
UV-Vis transmittance (**a**) and absorbance (**b**) in bulk and fibrous PS.

The UV-Vis optical spectra in transmittance and absorbance have been collected in the bulk and multiwire PS, and are shown in Figure 6a and Figure 6b, respectively.

The transmittance in the PS multiwire decreases from the NIR to the visible to the NUV regions, assuming values from zero at 250 nm to 9.8% at 600 nm and 11% at 850 nm. The transmittance in PS bulk decreases from the NIR to the visible to the NUV regions, assuming values that range from zero at 250 nm to 37% at 600 nm to 44% at 850 nm.

The absorbance in PS multiwire is nearly constant in the visible region and increases quickly in the UV region for wavelengths less than 300 nm. The same trend is observed for the PS bulk absorbance.

The absorbance in PS bulk is evaluated in a 30 μm thick foil, while that in PS multiwire is measured in a 250 μm foil; thus, it is not possible to have detailed information on the UV absorption due to PS, but from these it is possible to evaluate their average values over large wavelength regions. In the visible and IR regions, both absorbance spectra show a significant background signal due to the light scattering, which is more evident in the PS multiwire sample. In addition to the difference in density and thickness, this could be due to the different exposed polymer surfaces and intrinsic structure [17].

The distilled water wetting angle measurements have indicated that PS is hydrophobic; in fact, the contact angle is higher than 90°.

**Figure 7:**
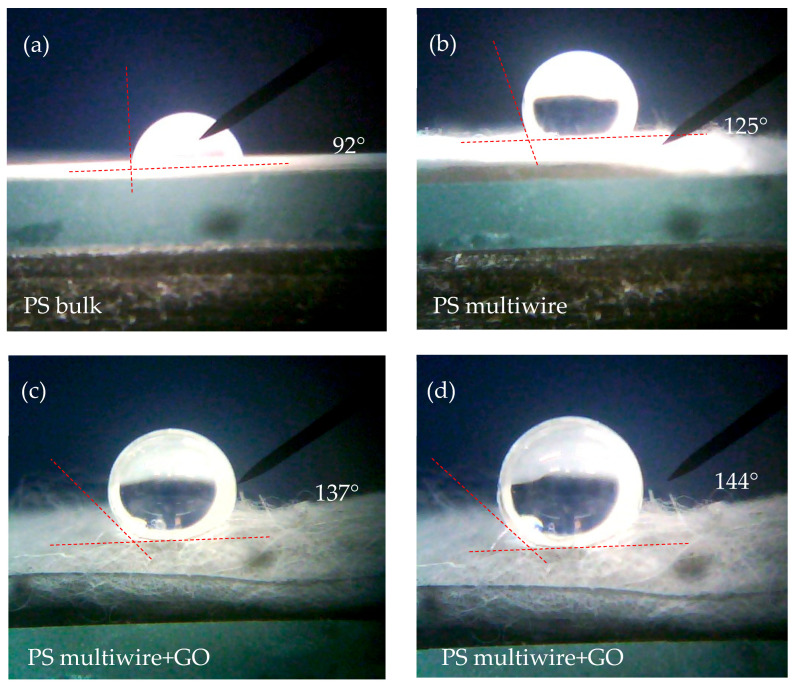
Contact angles for PS bulk (**a**), multiwire (**b**), and multiwire with 10 wt% GO NPs in two different areas (**c**,**d**).

Figure 7 reports the contact angle measurements for pure and bulk PS (a), the contact angle for the multiwire PS surface (b), and the contact angle for the multiwire PS with 10 wt% GO concentration embedded in two different areas (c,d). At room temperature, 1 atm pressure, and 50% humidity, the PS bulk has a contact angle of 92°, while the PS multiwire has an angle of 125°.

The use of GO embedded into the polymer significantly enhances the surface polymer hydrophobicity, which reaches a value between 137° and 144°, as visible in the photos in Figure 7c and Figure 7d, respectively.

Typically, the incorporation of GO in PS, due to its oxygen-containing functional groups, should increase the hydrophilicity of the composite and subsequently decrease the contact angle. However, in our case, an increase in contact angle was observed with the addition of GO. The inclusion of GO can be responsible for the increase in surface roughness and the formation of micro/nanoscale surface features. The Cassie–Baxter model [34] suggests that the presence of roughness/features can trap air and lead to an increase in the apparent contact angle, despite the intrinsic hydrophilicity of GO [35].

**Figure 8:**
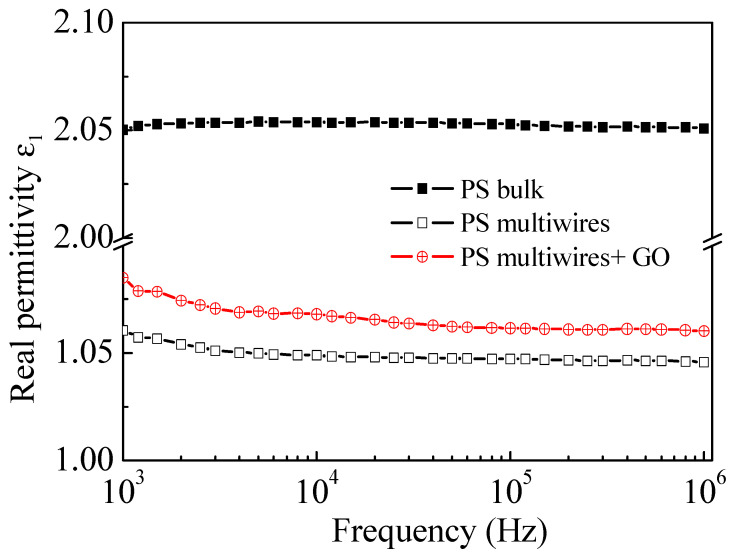
ε_1_ vs. frequency for polystyrene foil (PS bulk) and for multiwire PS without and with GO NPs at room temperature.

Regarding the dielectric response of the investigated PS samples, Figure 8 shows the frequency dependence of the real part of permittivity, ε_1_, for polystyrene (PS) in the form of both foil (PS bulk) and multiwire PS fiber. In the same figure, ε_1_ vs. frequency is also displayed for the multiwire PS containing GO NPs at the 10 wt% concentration. As one can see in this figure, the investigated frequency range for ε_1_ is frequency independent for the PS foil (PS bulk), with a value of about 2.05. When polystyrene is manufactured into fiber, the formation of pores with the consequent introduction of air, whose ε_1_ is equal to 1, into pores causes only a decrease in the ε_1_ value, which is now about 1.05, keeping its behavior almost unchanged as a function of frequency. The addition of GO NPs gives rise to a slight increase in the ε_1_ value, which has a slightly higher ε_1_, about 1.07, than multiwire PS.

**Figure 9:**
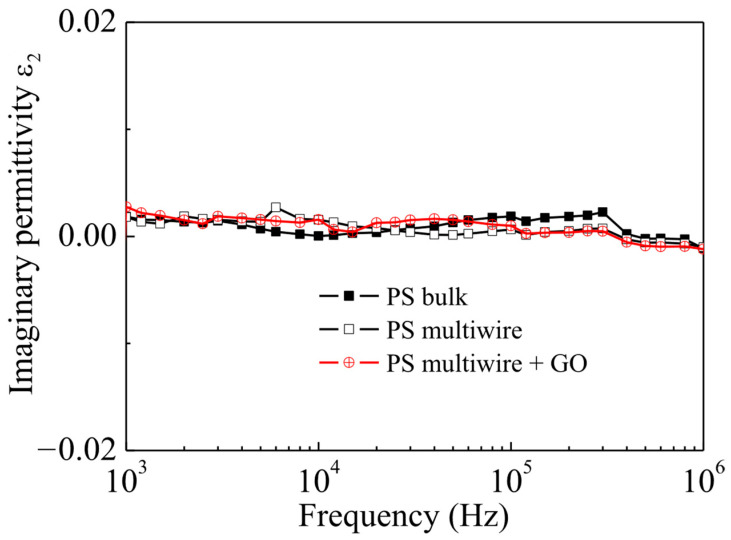
ε_2_ vs. frequency for polystyrene foil (PS bulk) and for multiwire PS without and with GO NPs at room temperature.

Also, the imaginary part of permittivity, ε_2_, called “dielectric loss factor”, of the three kinds of polystyrene is very close to zero, as shown in Figure 9. The observed trends in this study for ε_1_ and ε_2_ in the three investigated samples allow us to classify all three kinds of polystyrene as good dielectrics almost without losses.

**Figure 10:**
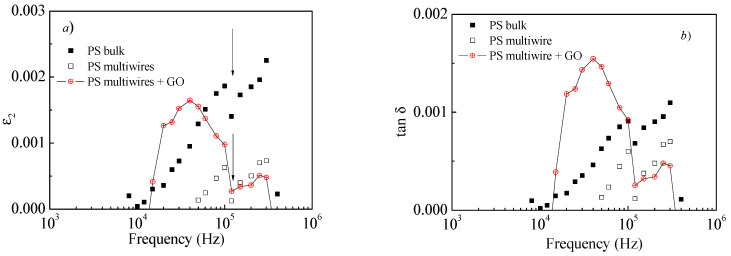
Zoom of Figure 9, where the arrows indicate instrumental artifacts (**a**) and the loss tangent tan δ of PS bulk; multiwire PS without and with GO NP samples (**b**) vs. frequency at room temperature.

A more accurate analysis of Figure 9, obtained expanding the vertical axes as shown in Figure 10a, has pointed out the presence of a relaxation process with a maximum in the imaginary component of the permittivity, ε_2_, which is well inside the measurement region for the multiwire PS + GO NP sample, but outside of the measuring range for the PS bulk and multiwire PS samples. The presence of this dielectric loss peak, located at about 40 kHz (see Figure 10a), is confirmed by the loss tangent tan δ vs. frequency plot, as illustrated in the successive Figure 10b, and its being visible only in the multiwire PS + GO NP sample could be due to the presence of GO in the polymer.

Thus, because the dielectric loss factor is very near zero, the electrical conductivity of the polymer is negligible and remains so also when a concentration of 10 wt% is embedded with GO NPs. The dielectric loss factor of polystyrene is very close to zero, suggesting that these materials cannot be candidates for electromagnetic wave absorption applications [9]. For the investigated composites, it seems that electromagnetic waves are not absorbed much. However, if reduced GO is employed as embedded conductive nanoparticles in the polymer, it is possible to obtain a significant absorption of microwaves, as reported in the literature [36].

The used GO nanoparticles are insulators and achieve significant electrical conductivity only when they are reduced through thermal or other processes that remove the various functional oxygen groups embedded in GO [37,38].

Other measurements concerning the property comparison between PS bulk, multiwire, and multiwire PS with the 10 wt% GO NP concentration have been performed by measuring the PS sample densities, water absorption, and air diffusion coefficient at room temperature. The first measurement uses a caliber and a microbalance to evaluate the mass density, obtaining densities comparable with the literature data [39]. The second one uses a microbalance and distilled water to measure the liquid absorbance, obtaining results comparable with the literature data [40]. The third one uses a high air pressure gradient applied to a known thickness of PS foil and evaluates the time to decrease the gradient to 1/*e* of the maximum value, as described in similar experiments reported in the literature [41].

The main results obtained in this investigation are reported as a comparison of PS bulk and multiwire without and with GO NPs, as shown in Table 2.

A final discussion concerns the possible applications of this polymer. Polystyrene is an important packaging material because it is light and protective, and, as a thin film or multiwire foil, it permits oxygen permeation or gas filtering. PS is used for food containers and trays [42]; protective packaging for electronics and fragile items; disposable plates and cutlery; thermal and electrical insulation panels; rigid and durable electronic housings and components; biology and medicine applications; medical device casings, test tubes and Petri dishes; sterile syringes and pipettes; and microfilters and sponges [43].

The multiwire PS finds more important applications, such as in desiccant cooling systems, in air dehumidification or humidification, in heat storage, in high water adsorbers at room temperature, in air treatment, in water desalination, in the filtering of toxic gases and liquids, and in others [44,45,46].

## 4. Conclusions

The electrospun polystyrene micro- and nanowires display significantly enhanced surface properties compared to bulk polystyrene. These high-aspect-ratio fibers, characterized by micro- or nanoscale diameters and extended lengths, exhibit remarkable surface effects such as the entrapment of both low- and high-molecular-weight molecules, variation in hydrophobicity or hydrophilicity, and the capacity to adsorb gases, nanoparticles, microparticles, and liquids. These features make multiwire PS particularly attractive for applications in sensing, filtration, membrane technology, and absorbent materials, where surface interactions are crucial. In this context, the integration of graphene oxide (GO) micro- and nanoparticles into electrospun PS fibers introduces further functional advantages. GO is rich in oxygen-containing functional groups (e.g., hydroxyl, carboxyl, epoxy), enabling additional mechanisms of molecular adsorption, gas capture, and water uptake. Furthermore, thermal or chemical treatment of GO embedded in PS at relatively high concentrations can promote partial reduction in GO, enhancing the electrical and thermal conductivity of the composite, while also modulating its interaction with specific molecular species. In this study, the innovative multiwire PS samples were investigated by means of various techniques to deduce information about morphology, the optical response in the UV, visible, and IR regions, the wetting ability, and other parameters such as density, water absorption between the fibers, and dielectric properties.

The results have evinced the low density and high porosity of the multiwire polymer with and without GO. Such polymers can absorb high quantities of gases, liquids, and nano- and microparticles between the wires, making the polymer highly effective for molecular absorbance uses. The absorption of many substances, in liquid and/or gas phase, may concern organic and inorganic species, acids and bases, and toxic and non-toxic molecules trapped between the stable PS microfibers, thanks to their very high measured hydrophobicity.

PS can be prepared as multiwire fibers to create a polymer useful for many applications in different fields, from industry to microelectronics and from medicine to biocompatible devices and food preservation methods. This innovative polymer has high porosity between the fibers and maintains its composition properties while remaining less rigid than bulk polystyrene. Its fiber diameter is about 1 micron, and its length is about 1 mm or more.

It is opaque and absorbs more visible and UV light than the bulk PS; consequently, it can be employed to realize optical absorbers and filters. The presence of embedded GO NPs enhances the fibrous PS hydrophobicity, increases the fibrous PS permittivity, and decreases the visible transmittance, producing a black-colored polymer concerning the white multiwire PS.

Multiwire PS does not become hydrophilic, as expected, due to the higher surface exposition and irregularities, but instead enhances its hydrophobicity properties even if GO NPs are embedded into it at high concentrations up to 10 wt%.

Its dielectric properties change due to a decrease in the relative permittivity of the multiwire concerning the PS bulk. The permittivity decreases significantly in multiwire PS both without and with the addition of GO NPs, but its electrical conductivity remains low, even if GO NPs are embedded into the multiwire PS.

The polymer may find many applications, especially for the absorption of liquids in high quantities, enhancing the pristine polymer’s weight by about 2000 wt%. Moreover, these polymers find applications for micro- and nanoparticles, liquid and gas filtration, oxygen permeation, and the possibility of being employed as composites in many materials, membranes, and sensor devices.

The goal of this study is to produce and investigate the properties of the PS-GO composite material, rather than to test a specific application. A thorough application-oriented study would require additional measurements and protocol development beyond the scope of the current manuscript. Nonetheless, we believe that the data presented here may serve as a solid foundation for future application studies in relevant fields.

## Figures and Tables

**Table 1:** Summary of electrospun samples and electrospinning parameters.

Sample Code	Weight Ratio Polymer/Solvent	Weight Ratio Additive/Polymer	Voltage (kV)	Flow Rate (µL/min)	Needle to Collector Distance (cm)
**PS**	21.9/78.1	-	12.5	15.0	15.0
**PS-GO**	21.9/78.1	10.0/90.0	12.0	15.0	15.0

**Table 2:** Some results of our experimental measurements concerning the comparison of properties between the PS bulk and multiwire without and with GO NPs.

Property	PS Bulk	Multiwire PS	Multiwire PS + GO 10 wt%
**Density** **(g/cm^3^)**	0.98–1.05	0.020	0.022
**Transmittance** **(550 nm)**	35% (30 μm)	9.5% (250 μm)	4% (250 μm)
**Contact angle** **(300 K, degrees)**	92	125	144
**Relative dielectric permittivity** **(100 KHz)**	2.05	1.05	1.06
**Water absorption** **(wt%)**	0	1900	1500
**Air diffusion coefficient** **(×10^8^ cm^2^/s)**	6	0.05	0.04

## Data Availability

The original contributions presented in this study are included in the article. Further inquiries can be directed to the corresponding author.
